# Variation in *GYS1* Interacts with Exercise and Gender to Predict Cardiovascular Mortality

**DOI:** 10.1371/journal.pone.0000285

**Published:** 2007-03-14

**Authors:** Jenny Fredriksson, Dragi Anevski, Peter Almgren, Marketa Sjögren, Valeriya Lyssenko, Joyce Carlson, Bo Isomaa, Marja-Riitta Taskinen, Leif Groop, Marju Orho-Melander, for the Botnia Study Group

**Affiliations:** 1 Department of Clinical Sciences in Malmö, Clinical Research Centre, Lund University, Malmö, Sweden; 2 Department of Mathematical Sciences, Chalmers University of Technology, Göteborg, Sweden; 3 Folkhälsan Genetic Institute, Folkhälsan Research Center, Biomedicum, Helsinki and Malmska Municipal Health Care Center and Hospital, Jakobstad, Finland; 4 Department of Medicine, Helsinki University Central Hospital, University of Helsinki, Finland; 5 Research Program of Molecular Medicine, University of Helsinki, Helsinki, Finland; North Carolina State University, United States of America

## Abstract

**Background:**

The muscle glycogen synthase gene (*GYS1*) has been associated with type 2 diabetes (T2D), the metabolic syndrome (MetS), male myocardial infarction and a defective increase in muscle glycogen synthase protein in response to exercise. We addressed the questions whether polymorphism in *GYS1* can predict cardiovascular (CV) mortality in a high-risk population, if this risk is influenced by gender or physical activity, and if the association is independent of genetic variation in nearby apolipoprotein E gene (*APOE*).

**Methodology/Principal Findings:**

Polymorphisms in *GYS1* (XbaIC>T) and *APOE* (-219G>T, ε2/ε3/ε4) were genotyped in 4,654 subjects participating in the Botnia T2D-family study and followed for a median of eight years. Mortality analyses were performed using Cox proportional-hazards regression. During the follow-up period, 749 individuals died, 409 due to CV causes. In males the *GYS1* XbaI T-allele (hazard ratio (HR) 1.9 [1.2–2.9]), T2D (2.5 [1.7–3.8]), earlier CV events (1.7 [1.2–2.5]), physical inactivity (1.9 [1.2–2.9]) and smoking (1.5 [1.0–2.3]) predicted CV mortality. The *GYS1* XbaI T-allele predicted CV mortality particularly in physically active males (HR 1.7 [1.3–2.0]). Association of *GYS1* with CV mortality was independent of *APOE* (219TT/ε4), which by its own exerted an effect on CV mortality risk in females (2.9 [1.9–4.4]). Other independent predictors of CV mortality in females were fasting plasma glucose (1.2 [1.1–1.2]), high body mass index (BMI) (1.0 [1.0–1.1]), hypertension (1.9 [1.2–3.1]), earlier CV events (1.9 [1.3–2.8]) and physical inactivity (1.9 [1.2–2.8]).

**Conclusions/Significance:**

Polymorphisms in *GYS1* and *APOE* predict CV mortality in T2D families in a gender-specific fashion and independently of each other. Physical exercise seems to unmask the effect associated with the *GYS1* polymorphism, rendering carriers of the variant allele less susceptible to the protective effect of exercise on the risk of CV death, which finding could be compatible with a previous demonstration of defective increase in the glycogen synthase protein in carriers of this polymorphism.

## Introduction

Cardiovascular (CV) disease (CVD), including coronary heart disease (CHD) and stroke, is the leading cause of death and disability in the Western world [1] and is thought to result from a complex interaction between genetic and environmental factors. Such risk factors are age, male gender, smoking, hypertension, diabetes, dyslipidemia [2] and physical inactivity. The genetic constitution of an individual usually determines how the individual responds to these risk factors. Therefore, it is necessary not only to identify which genetic variants increase susceptibility to the disease but also which environmental risk factors act in concert with these genes. In addition, the cellular environment in men and woman can be very different given known differences in hormonal milieu and gene expression [3]. Therefore, it is reasonable to consider the possibility that gender specific gene-environment interactions could modify the penetrance and expression of the trait.

Muscle glycogen synthase is the key enzyme in the synthesis of glycogen in skeletal muscle. A polymorphism (XbaI) in intron 14 of the glycogen synthase gene (*GYS1*) has been associated with lower glycogen synthase activity, T2D, features of the metabolic syndrome (MetS) and with myocardial infarction in males [4]–[8] but association to T2D has not been consistently replicated in all studies [9], [10]. Interestingly, electrical stimulation of skeletal muscle to mimic physical exercise increased the amount of glycogen synthase in carriers of wild-type C-allele but not in carriers of the T-allele. As a consequence, carriers of the T-allele may benefit less from physical exercise than carriers of the normal allele [11]. *GYS1* is located on chromosome 19q13.3, a region that has in several linkage studies been linked to MetS and T2D associated phenotypes [12]–[17]. Further, the *GYS1* locus was in the HERITAGE family study linked to glucose effectiveness in response to endurance exercise [18].

*GYS1* is separated only by 4.1 million base pairs from the gene coding for apolipoprotein E (*APOE*), which constitutes three common genetic isoforms in plasma and is known to play an important role in lipid metabolism [19]. The APOE4 isoform encoded by the ε4 allele is associated with elevated serum total- and low density lipoprotein (LDL)-cholesterol concentrations [20], [21] and with coronary heart disease (CHD) [22], [23]. In addition, a –219 (G>T) polymorphism in the *APOE* promoter has *in vitro* been shown to decrease transcriptional activity of *APOE* [24] and has been reported to associate with severity of coronary artery disease [25] and increased risk for myocardial infarction [26].

Given the considerations above, we set out to test 1) whether the *GYS1* polymorphism is associated with CV mortality in individuals from a large T2D family study from Finland, the Botnia Study. In particular we were interested in putative gender differences as the *GYS1* polymorphism has earlier been associated with myocardial infarction only in males, 2) whether physical exercise would act as an environmental factor interacting with the effect associated with the *GYS1* polymorphism as this has earlier been shown to be associated with defect in stimulation of glycogen synthase protein levels after muscle stimulation, and 3) to test if our results with the *GYS1* polymorphism are independent of the adjacent *APOE*. As the endpoint we used CV mortality after a median follow up period of 8 years.

## Methods

### Study Population

The Botnia Study was initiated in 1990 and represents a large population-based family study in Finland and Sweden aiming at identification of genes increasing susceptibility to T2D, MetS and related disorders. Details of the study cohort, sampling strategy as well as anthropometric and metabolic measurements have been described in detail [27], [28]. The study protocol was approved by the local ethics committees and an informed consent was obtained from each subject before participating in the study. The present study was restricted to the original Botnia cohort of 4654 subjects from 965 families (2142 males, 2512 females, age 58.2±13.8 years) from Western Finland. At the baseline examination, a structured questionnaire was completed by specially trained nurses, covering information about diseases other than T2D (particularly hypertension, coronary heart disease, myocardial infarction and stroke) and data on smoking habits and physical activity during work and leisure time. Both previous and current smokers were recorded as smokers. Physical activity level during work was defined on a scale from 0 to 6 according to level of physical activity (0 coding for no work and 6 for highest level) while physical activity during leisure time was estimated by a scale from 1 to 3 (1 = almost no activity at all; 2 = sometimes, but not regular; 3 = regular physical activity). Information on work and leisure time physical activity was combined to obtain an estimate of total physical activity level and classified as: 1) no physical activity or low physical activity (work level of 0 to 2 in combination of leisure time level of 1); 2) normal to high physical activity (work activity level 0–2 in combination of leisure time activity of >1; or work activity level ≥3 in combination of any leisure time activity level). When division between high and normal physical activity was needed, normal physical activity was defined as work activity level ≥3 and leisure time of <3 and high physical activity was defined as leisure time activity of 3 in combination with any work activity level. Glucose tolerance, assessed by an oral glucose tolerance test, and MetS were defined according to current World Health Organization (WHO) criteria [29]. Insulin resistance was estimated as the Homeostasis Model Assessment index (HOMA_IR_ = fasting serum insulin*fasting plasma glucose/22.5).

Total and CV mortality was assessed with median follow up time of 7.9 years and mortality data were obtained from central death-certificate registry in Finland. CV mortality was classified using the 9^th^ revision of the International Classification of Diseases (CV diagnosis codes 390–459) before 1997 and the 10^th^ revision (codes 100–199) thereafter. Causes of death were classified as 1) CV death (CHD, cerebrovascular disease (including both thrombotic stroke and cerebral haemorrhage) or other CV events (including pulmonary embolism, abdominal aortic aneurysm, hypertensive complications, general atherosclerosis and peripheral artery disease with gangrene) or 2) other causes of death (neoplasma, violent or other).

### Genotyping

A total of 4654 subjects were genotyped for the XbaI polymorphism in intron 14 (rs8103451) of *GYS1* and for the APOE isoforms encoded by amino acid substitutions at residues 112 (rs429358) and 158 (rs7412), for the –219G>T promoter polymorphism (rs405509). The XbaI polymorphism in *GYS1* was genotyped using single base pair extension on AB3100 (Applied Biosystems) and the *APOE* polymorphisms were genotyped using allelic discrimination on AB7900 at the SWEGENE DNA genotyping Laboratory. Before any analyses were performed, the expected risk-genotypes for *GYS1* and *APOE* were defined as CT or TT (*GYS1* XbaI), ε3ε4 or ε4ε4 (*APOE* codon 112 and 158 polymorphisms) and TT (*APOE* –219 polymorphism), respectively. Risk-alleles were defined according to previous T2D and MetS association study results for *GYS1* XbaI [4], [7], [8] and reports on *APOE* and risk of coronary disease [22], [25], [26]. To assure high quality of the produced genotypes, a random sample of 17.8% of all *GYS1* XbaI genotypes were repeated using PCR and restriction fragment length polymorphism and the concordance rate was 99.9% [30].

### Statistical Analysis

Allele- and genotype frequencies between groups were compared by the χ^2^ test or by Fisher's exact test whereas multiple regression was used to compare clinical variables between groups, adjusting for age, sex and BMI. Hardy-Weinberg equilibrium (HWE) was tested using exact test (http://ihg.gsf.de/cgi-bin/hw/hwa1.pl) with alpha level of <0.05 for rejection. For the survival analyses the data were treated as left truncated and right censored, meaning that age was the basic time variable. Survival curves were obtained with the Kaplan-Meier estimator, and nonparametric two-sample tests for genetic effects were performed with the log-rank test. Covariates from the baseline visit were used. Effects of genetic and clinical variables on survival time were analysed with uni- and multivariate Cox regression analyses, stratified for sex and using a robust variance estimate to adjust for within family dependence by treating each pedigree as an independent entity when calculating the variance. The univariate analyses were performed to obtain relevant set of variables for multivariate analyses, therefore these p-values were not corrected for multiple testing. The multivariate Cox models were obtained by stepwise forward inclusion of the covariates and statistical significance of the model was analysed using the Wald test. Individuals with missing data for any of the covariates were excluded from the analyses. Due to missing data on microalbuminuria (data missing for 35%) this variable was not included in the multivariate analysis.

Multiple tests were performed within the study (2 genes with 3 polymorphic sites and subanalyses in males and females). Concerning the XbaI polymorphism in males subanalyses were also performed according to physical activity level (low or normal to high). We did not correct for the number of analysed genes and polymorphisms as this study was designed to test the hypothesis that the T-allele of the *GYS1* XbaI polymorphism could be associated with CV mortality and as the *APOE* markers were studied to test if the *GYS1* results are independent of the adjacent *APOE*. For the gender-specific analyses and for the analyses in individuals with different physical activity levels we report both non-adjusted (p) and adjusted (p_c_) p-values. The gender-specific analyses were multiplied with a factor of 3 (3 groups; all, males, females) and the physical activity analyses with a factor of 6 (3 groups with either low or normal to high physical activity).

All statistical analyses were performed using Number Crunching Statistical Systems version 2004 (NCSS; Kaysville, Utah, USA) or R (www.r-project.org). Two sided p-values of less than 0.05 were considered statistically significant. Estimates of linkage disequilibrium were calculated using the Haploview program [31]. Power calculations were performed using the normal distributions for the coefficient estimates in the Cox regression model [32].

## Results

### Clinical and metabolic risk factors for CV mortality

During a median follow-up time of 7.9 years, 749 of the 4654 individuals (16.1%) had died and of them 409 (54.6%) due to CV causes (Table 1). Total mortality was slightly higher among males than among females (17.4 vs. 15.0%, p = 0.029), while frequency of CV mortality did not significantly differ between males and females (9.2 vs. 8.4%, p = 0.32). Subjects who died of CV causes had lower high density lipoprotein (HDL) cholesterol levels compared to both living subjects (p<0.0001) and individuals who died of other than CV causes (p = 0.0009). They also had higher triglyceride levels (p<0.0001) and higher frequency of T2D (p<0.0001), MetS (p = 0.0002), hypertension (p = 0.015), microalbuminuria (p<0.0001), earlier CV events (<0.0001) and lower physical activity level than subjects who were alive. CV death was associated with higher BMI (p = 0.046), total cholesterol levels (p = 0.0037), frequency of T2D (p<0.0001) and earlier CV events (p<0.0001) than death of other causes (Table 1).

**Table 1:** CHARACTERISTICS OF THE STUDY SUBJECTS

CHARACTERISTIC	ALL SUBJECTS	ALIVE	CV DEATH	OTHER DEATH
N (males/females)	4654 (2142/2512)	3905 (1770/2135)	409 (198/211)	340 (174/166)
Age (years)	58.2±13.8	55.3±12.5	75.0±9.1	71.6±11.0
BMI (kg/m^2^)	26.8±4.4	26.8±4.4	27.3±4.4	26.8±4.2
WH -males	0.96±0.06	0.96±0.06	0.97±0.07	0.97±0.07
-females	0.85±0.08	0.85±0.08	0.89±0.08	0.87±0.06
Cholesterol (mmol/l)	5.8±1.1	5.8±1.1	5.9±1.3	5.7±1.2
HDL cholesterol (mmol/l)	1.3±0.3	1.3±0.3	1.2±0.3	1.3±0.3
Triglycerides (mmol/l)	1.5±1.1	1.5±1.0	2.0±1.2	1.8±1.5
Type 2 diabetes (%)	34.3	27.5	78.6	60.3
Metabolic syndrome (%)	40.7	37.0	62.2	54.8
Hypertension (%)	47.2	43.2	71.9	64.1
Microalbuminuria (%)	7.7	6.1	22.8	15.8
Earlier CV events (%)	18.4	14.3	51.3	27.4
Smoking (%)	38.6	38.6	36.5	40.3
Low physical activity (%)	12.9	8.0	45.2	33.1

BMI; body mass index, WH; waist to hip ratio

Male gender, abdominal obesity, dyslipidaemia, T2D, hypertension, microalbuminuria, earlier CV events, smoking and low physical activity level were significant predictors of CV mortality among all individuals in univariate Cox regression analyses (Table 2). Gender specific univariate analyses identified low HDL cholesterol, T2D, hypertension, microalbuminuria, earlier CV events and physical inactivity as significant risk factors in both genders. Smoking was a significant risk factor only among male subjects while abdominal obesity and elevated triglyceride levels were significant predictors of CV death only in females (Table 2).

**Table 2:** CLINICAL AND GENETIC RISK FACTORS FOR CV MORTALITY

	ALL INDIVIDUALS		MALE SUBJECTS		FEMALE SUBJECTS	
	HR [95% CI]	P	HR [95% CI]	p	HR [95% CI]	p
Male sex	1.6 [1.3–1.9]	<0.0001				
BMI (kg/m^2^)	1.0 [1.0–1.0]	0.060	1.0 [1.0–1.1]	0.14	1.0 [1.0–1.1]	0.078
WH	27.6 [7.9–96.5]	<0.0001	3.7 [0.2–73.1]	0.39	18.8 [3.2–111.4]	0.0012
Cholesterol (mmol/l)	1.0 [0.9–1.1]	0.52	1.0 [0.9–1.2]	0.76	1.0 [0.9–1.2]	0.88
HDL-cholesterol (mmol/l)	3.1 [2.0–4.1]	<0.0001	2.5 [1.3–4.8]	0.0049	2.7 [1.7–4.1]	<0.0001
Triglycerides (mmol/l)	1.1 [1.1–1.2]	0.0017	1.1 [1.0–1.2]	0.16	1.4 [1.2–1.5]	<0.0001
Type 2 diabetes	3.2 [2.5–4.2]	<0.0001	3.2 [2.3–4.6]	<0.0001	3.2 [2.2–4.8]	<0.0001
Metabolic syndrome	1.3 [1.0–1.5]	0.030	1.3 [1.0–1.7]	0.10	1.2 [0.9–1.6]	0.25
Hypertension	1.4 [1.1–1.7]	0.0046	1.4 [1.1–1.9]	0.021	1.4 [1.0–2.0]	0.036
Microalbuminuria	2.3 [1.6–3.3]	<0.0001	2.1 [1.3–3.3]	0.0014	2.3 [1.4–4.1]	0.0022
Earlier CV events	2.5 [2.0–3.0]	<0.0001	2.8 [2.0–3.7]	<0.0001	2.1 [1.6–2.8]	<0.0001
Smoking	1.7 [1.3–2.1]	<0.0001	1.5 [1.1–2.1]	0.0075	1.1 [0.5–2.2]	0.83
Low physical activity	2.6 [2.0–3.3]	<0.0001	2.9 [2.1–4.0]	<0.0001	2.6 [1.9–3.6]	<0.0001
*APOE* E3E4/E4E4	1.1 [0.9–1.4]	0.31	0.9 [0.6–1.2]	0.43	1.4 [1.0–1.9]	0.030
*APOE* –219 TT	1.1 [0.9–1.4]	0.32	0.8 [0.5–1.1]	0.19	1.5 [1.1–2.1]	0.0082
*APOE* risk genotype combination	1.3 [1.0–1.8]	0.064	0.6 [0.3–1.2]	0.14	2.3 [1.6–3.2]	<0.0001
*GYS1* XbaI CT/TT	1.2 [0.9–1.6]	0.24	1.8 [1.2–2.6]	0.0016	0.7 [0.4–1.2]	0.18

Univariate Cox proportional-hazards analysis, performed with robust variance estimate to adjust for within family dependence. BMI; body mass index, WH; waist to hip ratio

In multivariate analyses T2D, elevated fasting insulin concentration, earlier CV events, low physical activity and smoking were significant risk factors for CV mortality in males (model 1 in Table 3). In females T2D, high fasting glucose concentration, hypertension, earlier CV events, and physical inactivity were significant risk factors for CV mortality (model 1 in Table 4). Due to lack of data for a large part (35%) of the study subjects, microalbuminuria was not included in the multivariate model.

**Table 3:** MULTIVARIATE MODEL OF RISK FACTORS FOR CV MORTALITY IN MALES

RISK PHENO-/GENOTYPE	MODEL 1 CLINICAL VARIABELES	P	MODEL 2 CLINICAL AND GENETIC VARIABLES	P
*GYS1* XbaI (T)			1.9 [1.2–2.9]	3.5e^−3^ *
T2D	2.4 [1.6–3.7]	3.0e^−5^	2.5 [1.7–3.8]	1.2e^−5^
Fasting serum insulin	1.0 [1.0–1.0]	3.5e^−2^		
Earlier CV events	1.9 [1.4–2.7]	2.1e^−4^	1.7 [1.2–2.5]	6.0e^−3^
Low physical activity	1.9 [1.3–2.8]	1.7e^−3^	1.9[1.2–2.9]	3.1e^−3^
Smoking	1.6 [1.0–2.3]	3.4e^−2^	1.5 [1.0–2.3]	3.2e^−2^
*P*-value (model, Wald test)		3.9e^−14^		7.0e^−11^

Multivariate Cox regression analysis using stepwise forward inclusion with robust variance estimates. Adjusted for age, sex and family correlations. * P_c_ = 0.018

**Table 4:** MULTIVARIATE MODEL OF RISK FACTORS FOR CV MORTALITY IN FEMALES

RISK PHENO-/GENOTYPE	MODEL 1 CLINICAL VARIABELES	P	MODEL 2 CLINICAL AND GENETIC VARIABLES	P
*APOE* (E3E4/E4E4 and –219 TT)			2.9 [1.9–4.4]	2.6e^−6^ *
T2D	1.7 [1.0–2.9]	3.9e^−2^		
Fasting plasma glucose	1.1 [1.1–1.2]	1.3e^−5^	1.2 [1.1–1.2]	2.3e^−10^
BMI			1.0 [1.0–1.1]	2.2e^−2^
Hypertension	1.6 [1.1–2.4]	2.9e^−2^	1.9 [1.2–3.1]	7.3e^−3^
Earlier CV events	1.6 [1.1–2.3]	1.2e^−2^	1.9 [1.3–2.8]	5.9e^−4^
Low physical activity	2.1 [1.4–3.1]	2.5e^−4^	1.9 [1.2–2.8]	4.9e^−3^
*P*-value (model, Wald test)		5.4e^−14^		<1.0e^−10^

Multivariate Cox regression analysis using stepwise forward inclusion with robust variance estimates. Adjusted for age, sex and family correlations. * P_c_ = 7.8e^−6^

### Allelic association between the *GYS1* and *APOE* polymorphisms

Genotype frequencies of the *GYS1* XbaI polymorphisms and *APOE* in the study population were: *GYS1* XbaI C/T (CC 88.0%, CT 11.5%, TT 0.6%), *APOE* –219G>T (GG 29.5%, GT 50.2%, TT 20.3%), and *APOE* ε2/ε3/ε4 (ε2ε2 0.4%, ε2ε3 9.4%, ε2ε4 2.3%, ε3ε3 59.4%, ε3ε4 25.2%, ε4ε4 3.2%). The genotypes of all single nucleotide polymorphisms (SNPs) (*APOE* Cys112Arg, Arg158Cys, -219G>T and *GYS1* XbaI) and the relative frequencies of ε-alleles, were in Hardy Weinberg equilibrium in the whole study population. Neither the genotype frequencies nor their combinations differed between males and females. The *APOE* –219G>T and Arg158Cys as well as the Arg158Cys and Cys112Arg polymorphisms were in complete linkage disequilibrium (D′ = 1.0). The *GYS1* XbaI polymorphism was not in linkage disequilibrium with any of the three *APOE* SNPs (r^2^ = 0.0, for all and D′ = 0.12, 0.07 and 0.16, for *APOE*-219G>T, Cys112Arg and Arg158Cys, respectively).

### *GYS1* XbaI as a genetic predictor for CV mortality

**Figure 1:**
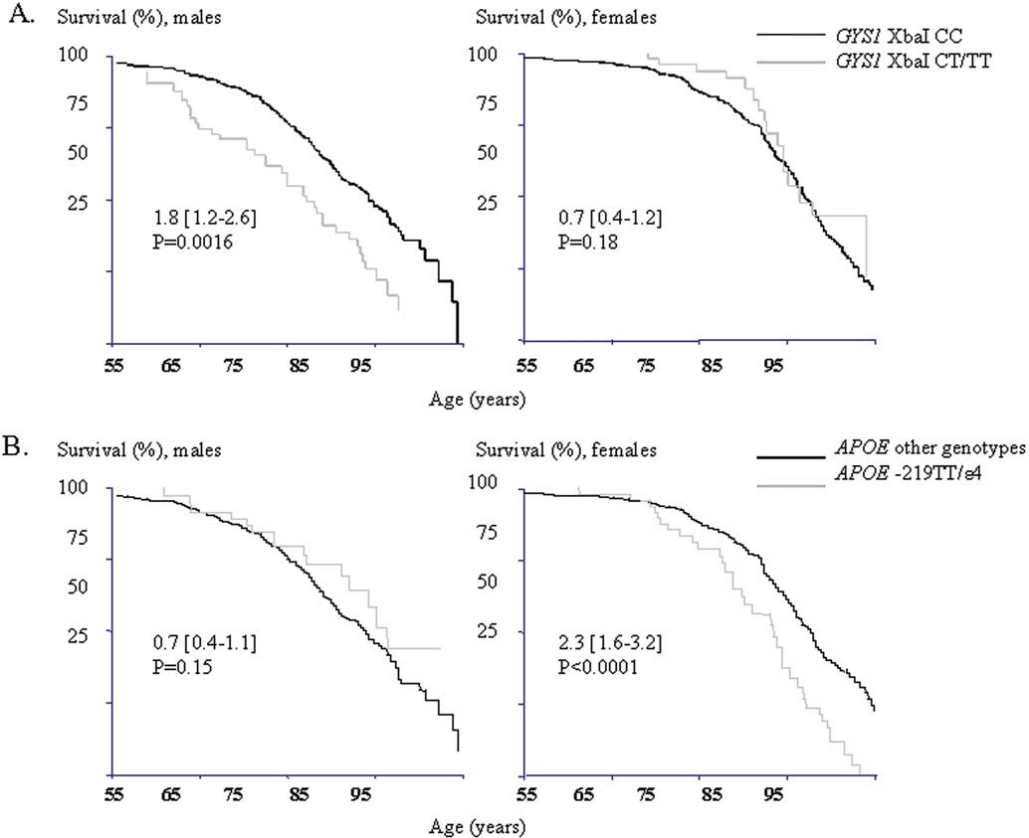
CV mortality in males and females according to the *GYS1* XbaI (A) and *APOE* –219/ε2/ε3/ε4 (B) genotypes. Kaplan Meier survival curves illustrating a higher risk for CV mortality (HR 1.8 [1.2–2.6], p = 0.0016, p_c_ = 0.0096) in male carriers of the *GYS1* XbaI CT/TT-genotypes and in female carriers of the *APOE* –219TT/ε4 genotype combination (HR 2.3 [1.6–3.2], p<0.0001, p_c_<0.0001).

The frequency of the XbaI risk genotypes (CT or TT) did not significantly differ between patients who died of CV causes, other causes or survivors when all subjects were included in the analyses (13.3%, 10.5%, and 12.0%) (Table 5). However, in gender-specific analyses, males with CV death had more often the CT/TT genotypes compared to surviving males (19.2 vs. 11.8%, p = 0.0038, p_c_ = 0.011). Consequently, in the Cox regression analysis, the *GYS1* XbaI CT/TT genotypes were significant predictors of CV mortality in males (Table 2 and Figure 1A). The CT/TT genotypes did not predict mortality due to other than CV causes.

**Table 5:** GENOTYPE DISTRIBUTION IN SUBJECTS WHO DIED FROM CV CAUSES AND IN SUBJECTS WHO ARE ALIVE OR DIED DUE TO OTHER CAUSES

GENE AND RISK GENOTYPE		CV DEATH	OTHER SUBJECTS
All subjects (N = 4654)		Percent	
*APOE* ε2/ε3/ε4	E3E4/E4E4	27.2	28.6
*APOE* -219	TT	23.1	20.1
*APOE* ε2/ε3/ε4/-219 risk genotype combination		12.8	10.2
*GYS1* Xba1	CT/TT	13.3	11.9
Males (N = 2142)			
*APOE* ε2/ε3/ε4	E3E4/E4E4	22.7	29.1
*APOE* -219	TT	19.1	20.8
*APOE* ε2/ε3/ε4/-219 risk genotype combination		7.7	11.2
*GYS1* Xba1	CT/TT	19.1^†^	11.8
Females (N = 2512)			
*APOE* ε2/ε3/ε4	E3E4/E4E4	31.4	28.2
*APOE* -219	TT	26.9^*^	19.5
*APOE* ε2/ε3/ε4/-219 risk genotype combination		17.5^‡^	9.4
*GYS1* Xba1	CT/TT	7.9	12.0

Fischer's exact test, *p = 0.019 (p_c_ = 0.057), †p = 0.0038 (p_c_ = 0.011), ‡p = 0.00048 (p_c_ = 0.0014)

Furthermore, when analysed together the *GYS1* XbaI polymorphism, T2D, earlier CV events, low physical activity and smoking were significant risk factors for CV mortality in males (model 2 in table 3).

### Does physical activity influence the effect associated with the genetic variation in *GYS1* on CV mortality risk?

**Figure 2:**
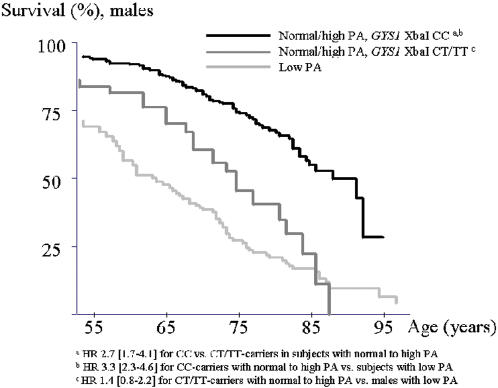
Interaction between the *GYS1* XbaI polymorphism and physical activity (PA) in males. Kaplan Meier survival curves for males reporting normal to high physical activity (PA) level according to *GYS1* XbaI genotype compared to males with low PA level.

CV mortality was significantly higher among individuals with low physical activity level compared to individuals with normal (29.9 vs. 7.1%, p<0.0001, corrected for sex and age) or high (29.9 vs. 5.7%, p<0.0001) physical activity level. The difference was not significant between groups reporting normal or high physical activity level (7.1 vs. 5.4%, p = 0.35) suggesting minimal or no protective effect above a normal level of physical activity on CV mortality risk. In a multivariate Cox regression analysis both physical activity (hazard ratio (HR) 3.2 [2.2–4.6], p<0.0001, p_c_<0.0001) and the XbaI polymorphism (HR 2.6 [1.7–3.8], p<0.0001, p_c_<0.0001) were strongly associated with CV mortality. While physical activity itself (normal or high) had a strong protective effect on CV mortality, this effect was attenuated in carriers of the CT/TT-genotypes of the XbaI polymorphism; physically active males with the CT/TT genotypes had a 2.7-times higher risk for CV mortality compared to CC-genotype carriers (HR 2.7 [1.8–4.1], p<0.0001, p_c_<0.0001) (Figure 2).

### *APOE* polymorphisms as genetic predictors for CV mortality

The frequency of the *APOE* ε2/ε3/ε4 risk genotypes (ε3ε4 or ε4ε4), the *APOE* –219 risk genotype (TT) or the risk genotype combination of *APOE*ε and *APOE* –219 (-219TT/ε4) did not significantly differ between individuals who died of CV causes and other subjects (Table 5). However, females in the CV mortality group had more often the *APOE* –219 TT-genotype, in particular -219TT/ε4 compared to surviving females (26.9 vs. 19.8%, p = 0.019, p_c_ = 0.057 and 17.5 vs. 9.4%, p = 0.00048, p_c_ = 0.0014). No effect of the *APOE* variants was observed in males, this effect being restricted to females in whom both the *APOE* ε4-allele, the –219 TT-genotype and their combination were significant predictors of CV mortality (Table 2 and Figure 1B), but not of non-CV mortality. When genetic and non-genetic factors were included in the analysis of risk of CV death, the *APOE* risk genotype combination, high fasting glucose level, high BMI, hypertension, earlier CV events and low physical activity were significant risk factors for CV mortality in females (model 2 in table 4).

### Cholesterol levels and CV mortality according to *APOE* ε3ε4/ε4ε4 and -219 TT genotypes

The *APOE* ε3ε4/ε4ε4 and -219 TT genotypes (and their combination) were associated with increased total and LDL cholesterol levels in both males and females (after adjustment for age, T2D and BMI). Interestingly, although the *APOE* ε3ε4/ε4ε4 variants had a statistically weaker effect on total cholesterol in female carriers (6.1±1.2 vs. 5.8±1.2 mmol/l, p = 0.014, p_c_ = 0.084 for the ε3ε4/ε4ε4 genotype vs. other genotypes) compared to non-carriers (6.1±1.2 vs. 5.8±1.1 mmol/l, p = 0.0001 p_c_ = 0.0006) of *APOE* -219 TT, they only predicted CV mortality in carriers of *APOE* -219 TT (HR 2.3, [1.3–4.2], p = 0.0059, p_c_ = 0.035). The -219 polymorphism had no effect on cholesterol levels neither in female carriers nor non-carriers of the *APOE* ε3ε4/ε4ε4, and predicted CV mortality only in carriers of the *APOE* ε3ε4/ε4ε4 genotypes (HR 2.3 [1.5–3.6], p = 0.00038, p_c_ = 0.0023).

In males, the *APOE* ε3ε4/ε4ε4 genotypes affected total cholesterol in carriers (5.9±1.0 vs. 5.6±1.2 mmol/l, p = 0.031, for the ε3ε4/ε4ε4 genotype vs. other genotypes, respectively) but not after correcting for multiple testing (p_c_ = 0.19) and not in non-carriers (5.7±1.0 vs. 5.6±1.1 mmol/l, p = 0.31) of the *APOE* -219 TT genotype. As in females, the *APOE* -219 TT genotype had no significant effect on cholesterol values neither in carriers nor in non-carriers of the *APOE* ε3ε4/ε4ε4 genotypes. In contrast to females, neither *APOE* ε3ε4/ε4ε4 nor *APOE* -219 TT predicted CV mortality in males.

### Independency between *GYS1* and *APOE* as risk factors for CV mortality

To investigate whether the ‘at-risk’ genotypes of *GYS1* and *APOE* contributed independently to the CV mortality risk, we performed Cox regression analyses by entering both genes into the equation. These analyses clearly indicated that the effect of *GYS1* XbaI CT/TT in males (XbaI CT/TT: HR 1.9 [1.3–2.7], *APOE* –219/ε4: HR 1.5 [0.9–2.5]), as well as the effect of *APOE* genotype combination in females (*APOE* –219/ε4: HR 2.4 [1.7–3.6], *GYS1* XbaI CT/TT: HR 1.3 [0.8–2.3]), were independent of each other. To further assess the independence of the effects of the polymorphisms on CV mortality, the samples were stratified according to *GYS1* and *APOE* genotypes: The XbaI T-allele was associated with CV mortality in males without the *APOE* risk genotype combination (HR 1.9 [1.3–2.8]) and the *APOE* risk-genotype combination was associated with cardiovascular mortality among female XbaI CC-carriers (HR 2.3 [1.6–3.5]).

## Discussion

The key findings of the present study were that 1) the XbaI polymorphism in *GYS1* was associated with CV mortality in males; 2) although physical activity markedly reduces risk of CV death, this protective effect was attenuated in male carriers of the XbaI polymorphism; and 3) despite the fact that *GYS1* is adjacent to *APOE* on chromosome 19q13, the effect of the *GYS1* polymorphism on CV mortality is independent of the effect of *APOE*, which exerts a strong effect on CV mortality risk by its own. Interestingly, this risk seems to be restricted to females and cannot fully be explained by the effect of the *APOE* alleles on cholesterol levels.

Several studies performed in different ethnic populations have reported linkage to chromosome 19q13 for LDL-cholesterol- [33]–[37] or triglyceride levels [38], obesity [39], [40], as well as for insulin resistance and T2D related phenotypes [12]–[17] but the underlying genetic variants have not been identified. In addition, glucose effectiveness in response to exercise training as well as significant sex specific differences in heritability models and sex interaction for HDL cholesterol have been mapped to the 19q13 region [18], [41]. We therefore set out to study the contribution of two candidate genes in this region, *GYS1* and *APOE* to CV mortality risk, focusing particularly on the role of putative interaction between *GYS1* polymorphism and gender and/or physical activity level to affect the CV mortality rate.

The *GYS1* XbaI polymorphism was significantly associated with increased risk for CV mortality in males, a result supported by our previous independent finding of an association between myocardial infarction and this particular polymorphism only in males in another study population [7].

As anticipated, a low physical activity level was a severe risk factor for CV mortality. The novel finding of our study was that the protective effect of physical exercise was attenuated in carriers of the XbaI polymorphism. This goes along with the hypothesis advanced by a Canadian study [11] that carriers of the risk T-allele have a defect in their ability to increase the glycogen synthase protein in response to neuromuscular electrical stimulation (as a proxy for physical exercise) [11]. An increase in glycogen synthase protein would promote glycogen formation which, in turn, could have a beneficial effect on exercise capacity. The downside of this message is that all individuals would not respond to physical exercise in the same way. The positive message is that the “non-responder” group is relatively small (frequency of CT/TT genotypes in the population is only 12%) and in 88% of the population exercise exerts a highly beneficial and protective effect on risk of CVD.

We have no apparent explanation for why the effect of the XbaI polymorphism was restricted to males. One potential explanation could be that women have less muscle mass and muscular strength than men, but also a tendency to metabolise fat rather than carbohydrate during exercise [42]. Moreover, women seem less vulnerable to exercise-induced sudden death [42]. A potential explanation could be that both exercise training and oestrogen increase Akt phosphorylation and glycogen synthase kinase-3 inactivation leading to increased glycogen synthase activity. Interestingly, markedly higher myocardial Akt nuclear activity has been reported in females than in males as well as in pre- compared to post-menopausal woman [43]. If this also applies to skeletal muscle it could provide a potential explanation for the observed gender-specific effect.

Our results are in agreement with the earlier results reporting either the *APOE* ε4-allele or the *APOE* -219 TT-genotype as risk factors for CVD and/or mortality [22], [23], [25], [26] but, interestingly, we could observe this effect only in females. Caution is, however, warranted in the interpretation of the gender-specific effects of *APOE* as the study included a large number of patients with T2D. It is known, that men with T2D have an excess mortality compared with women with T2D resulting in a relative increase in the frequency of female T2D patients with aging. It is therefore still possible that the *APOE* polymorphisms had an effect in male T2D resulting in premature death. A sub-analysis of men and women divided by the median of age did, however, not support such an explanation. Also, power is reduced when the analysis is restricted to gender. The power in a Cox regression analysis depends among other things on the accrual time during which patients are recruited, mean time to failure and the expected effect sizes (http://biostat.mc.vanderbilt.edu/twiki/bin/view/Main/PowerSampleSize)[44]. We used the standard Normal theory to calculate the power to detect a HR of a certain size [32]. Our study had a 73–96% power to detect hazard ratios of 1.5 for the analysed polymorphisms.

In conclusion, we demonstrate a protective effect of physical activity on CV mortality. However, in male subjects this effect was attenuated in carriers of the rare allele of the XbaI polymorphism in *GYS1*. This finding could be compatible with a previous demonstration of defective increase in the glycogen synthase protein in carriers of this polymorphism. We could exclude that the association between the *GYS1* polymorphism and CV mortality was due to the adjacent *APOE* gene. Instead, we demonstrated that this gene exerted an increased risk of CV mortality in females. These findings re-emphasize the need to consider the effect of genetic variants in complex diseases in concert with their environmental triggers but also to evaluate whether females and males respond differently to genes and the environment.

## References

[1] Bethesda (2002). National Heart, Lung and Blood Institute. NHLBI morbidity and mortality chartbook.. http://www.nhlbi.nih.gov/resources/docs/cht-book.htm.

[2] (1999). Summary of the second report of the National Education Program (NCEP) Expert Panel on Detection, Evaluation, and Treatment of High Blood Cholesterol in Adults (Adult treatment panel II).. JAMA.

[3] Rinn JL, Snyder M (2005). Sexual dimorphism in mammalian gene expression.. Trends Genet.

[4] Groop LC, Kankuri M, Schalin Jantti C, Ekstrand A, Nikula Ijas P (1993). Association between polymorphism of the glycogen synthase gene and non-insulin-dependent diabetes mellitus.. N Engl J Med.

[5] Zouali H, Velho G, Froguel P (1993). Polymorphism of the glycogen synthase gene and non-insulin-dependent diabetes mellitus.. N Engl J Med.

[6] Schalin-Jäntti C, Harkonen M, Groop LC (1995). Impaired activation of glycogen synthase in people at increased risk for developing NIDDM.. Diabetes.

[7] Orho-Melander M, Almgren P, Kanninen T, Forsblom C, Groop LC (1999). A paired-sibling analysis of the XbaI polymorphism in the muscle glycogen synthase gene.. Diabetologia.

[8] Fenger M, Poulsen P, Beck-Nielsen H, Vaag A (2000). Impact of the Xba1-polymorphism of the human muscle glycogen synthase gene on parameters of the insulin resistance syndrome in a Danish twin population.. Diabet Med.

[9] Kadowaki T, Kadowaki H, Yazaki Y (1993). Polymorphism of the glycogen synthase gene and non-insulin-dependent diabetes mellitus.. N Engl J Med.

[10] Rissanen J, Pihlajamaki J, Heikkinen S, Kekalainen P, Mykkanen L (1997). New variants in the glycogen synthase gene (Gln71His, Met416Val) in patients with NIDDM from eastern Finland.. Diabetologia.

[11] St-Onge J, Joanisse DR, Simoneau JA (2001). The stimulation-induced increase in skeletal muscle glycogen synthase content is impaired in carriers of the glycogen synthase XbaI gene polymorphism.. Diabetes.

[12] Pratley RE, Thompson DB, Prochazka M, Baier L, Mott D (1998). An autosomal genomic scan for loci linked to prediabetic phenotypes in Pima Indians.. J Clin Invest.

[13] Watanabe RM, Ghosh S, Langefeld CD, Valle TT, Hauser ER (2000). The Finland-United States investigation of non-insulin-dependent diabetes mellitus genetics (FUSION) study. II. An autosomal genome scan for diabetes-related quantitative-trait loci.. Am J Hum Genet.

[14] Mori Y, Otabe S, Dina C, Yasuda K, Populaire C (2002). Genome-wide search for type 2 diabetes in Japanese affected sib-pairs confirms susceptibility genes on 3q, 15q, and 20q and identifies two new candidate Loci on 7p and 11p.. Diabetes.

[15] Panhuysen CI, Cupples LA, Wilson PW, Herbert AG, Myers RH (2003). A genome scan for loci linked to quantitative insulin traits in persons without diabetes: the Framingham Offspring Study.. Diabetologia.

[16] van Tilburg JH, Sandkuijl LA, Strengman E, van Someren H, Rigters-Aris CA (2003). A genome-wide scan in type 2 diabetes mellitus provides independent replication of a susceptibility locus on 18p11 and suggests the existence of novel Loci on 2q12 and 19q13.. J Clin Endocrinol Metab.

[17] An P, Freedman BI, Hanis CL, Chen YD, Weder AB (2005). Genome-wide linkage scans for fasting glucose, insulin, and insulin resistance in the National Heart, Lung, and Blood Institute Family Blood Pressure Program: evidence of linkages to chromosome 7q36 and 19q13 from meta-analysis.. Diabetes.

[18] An P, Teran-Garcia M, Rice T, Rankinen T, Weisnagel SJ (2005). Genome-wide linkage scans for prediabetes phenotypes in response to 20 weeks of endurance exercise training in non-diabetic whites and blacks: the HERITAGE Family Study.. Diabetologia.

[19] Mahley R (1988). Apolipoprotein E: cholesterol transport protein with expanding role in cell biology.. Science.

[20] Sing CF DJ (1985). Role of apolipoprotein E polymorphism in determining normal plasma lipid and lipoprotein variation.. Am J Hum Genet.

[21] Ehnholm C, Lukka M, Kuusi T, Nikkilä E, Uterman G (1986). Apolipoprotein E polymorphisms in the Finnish population: gene frequencies and relation to lipoprotein concentrations.. J Lipid Res.

[22] Wilson PW, Schaefer EJ, Larson MG, Ordovas JM (1996). Apolipoprotein E alleles and risk of coronary disease. A meta-analysis.. Arterioscler Thromb Vasc Biol.

[23] Song Y, Stampfer MJ, Liu S (2004). Meta-analysis: apolipoprotein E genotypes and risk for coronary heart disease.. Ann Intern Med.

[24] Artiga MJ, Bullido MJ, Sastre I, Recuero M, Garcia MA (1998). Allelic polymorphisms in the transcriptional regulatory region of apolipoprotein E gene.. FEBS Lett.

[25] Ye S, Dunleavey L, Bannister W, Day LB, Tapper W (2003). Independent effects of the -219 G>T and epsilon 2/ epsilon 3/ epsilon 4 polymorphisms in the apolipoprotein E gene on coronary artery disease: the Southampton Atherosclerosis Study.. Eur J Hum Genet.

[26] Lambert JC, Brousseau T, Defosse V, Evans A, Arveiler D (2000). Independent association of an APOE gene promoter polymorphism with increased risk of myocardial infarction and decreased APOE plasma concentrations-the ECTIM study.. Hum Mol Genet.

[27] Groop L, Forsblom C, Lehtovirta M, Tuomi T, Karanko S (1996). Metabolic consequences of a family history of NIDDM (the Botnia study): evidence for sex-specific parental effects.. Diabetes.

[28] Isomaa B, Almgren P, Tuomi T, Forsen B, Lahti K (2001). Cardiovascular morbidity and mortality associated with the metabolic syndrome.. Diabetes Care.

[29] Alberti KG, Zimmet PZ (1998). Definition, diagnosis and classification of diabetes mellitus and its complications. Part 1: diagnosis and classification of diabetes mellitus provisional report of a WHO consultation [see comments].. Diabet Med.

[30] Schalin-Jantti C, Nikula-Ijas P, Huang X, Lehto M, Knudsen P (1996). Polymorphism of the glycogen synthase gene in hypertensive and normotensive subjects.. Hypertension.

[31] Barrett JC, Fry B, Maller J, Daly MJ (2005). Haploview: analysis and visualization of LD and haplotype maps.. Bioinformatics.

[32] Casella G, Berger, R L (1990). Statistical Inference:.

[33] Ober C, Abney M, McPeek MS (2001). The genetic dissection of complex traits in a founder population.. Am J Hum Genet.

[34] Beekman M, Heijmans BT, Martin NG, Whitfield JB, Pedersen NL (2003). Evidence for a QTL on chromosome 19 influencing LDL cholesterol levels in the general population.. Eur J Hum Genet.

[35] Bosse Y, Chagnon YC, Despres JP, Rice T, Rao DC (2004). Genome-wide linkage scan reveals multiple susceptibility loci influencing lipid and lipoprotein levels in the Quebec Family Study.. J Lipid Res.

[36] Adeyemo AA, Johnson T, Acheampong J, Oli J, Okafor G (2005). A genome wide quantitative trait linkage analysis for serum lipids in type 2 diabetes in an African population.. Atherosclerosis.

[37] Heijmans BT, Beekman M, Putter H, Lakenberg N, van der Wijk HJ (2005). Meta-analysis of four new genome scans for lipid parameters and analysis of positional candidates in positive linkage regions.. Eur J Hum Genet.

[38] Pollin TI, Hsueh WC, Steinle NI, Snitker S, Shuldiner AR, Mitchell BD (2004). A genome-wide scan of serum lipid levels in the Old Order Amish.. Atherosclerosis.

[39] Bell CG, Benzinou M, Siddiq A, Lecoeur C, Dina C (2004). Genome-wide linkage analysis for severe obesity in french caucasians finds significant susceptibility locus on chromosome 19q.. Diabetes.

[40] Feitosa MF, Rice T, North KE, Kraja A, Rankinen T (2005). Pleiotropic QTL on chromosome 19q13 for triglycerides and adiposity: The HERITAGE family study.. Atherosclerosis.

[41] Weiss LA, Pan L, Abney M, Ober C (2006). The sex-specific genetic architecture of quantitative traits in humans.. Nat Genet.

[42] Shephard RJ (2000). Exercise and training in women, Part I: Influence of gender on exercise and training responses.. Can J Appl Physiol.

[43] Camper-Kirby D, Welch S, Walker A, Shiraishi I, Setchell KD (2001). Myocardial Akt activation and gender: increased nuclear activity in females versus males.. Circ Res.

[44] Dupont W, Plummer Jr W PS: Power and Sample Size Calculation.

